# HPV16 oncogene expression levels during early cervical carcinogenesis are determined by the balance of epigenetic chromatin modifications at the integrated virus genome

**DOI:** 10.1038/onc.2016.8

**Published:** 2016-02-15

**Authors:** I J Groves, E L A Knight, Q Y Ang, C G Scarpini, N Coleman

**Affiliations:** 1 Department of Pathology, University of Cambridge, Cambridge, UK

## Abstract

In cervical squamous cell carcinomas, high-risk human papillomavirus (HRHPV) DNA is usually integrated into host chromosomes. Multiple integration events are thought to be present within the cells of a polyclonal premalignant lesion and the features that underpin clonal selection of one particular integrant remain poorly understood. We previously used the W12 model system to generate a panel of cervical keratinocyte clones, derived from cells of a low-grade premalignant lesion naturally infected with the major HRHPV type, HPV16. The cells were isolated regardless of their selective advantage and differed only by the site of HPV16 integration into the host genome. We used this resource to test the hypothesis that levels of HPV16 E6/E7 oncogene expression in premalignant cells are regulated epigenetically. We performed a comprehensive analysis of the epigenetic landscape of the integrated HPV16 DNA in selected clones, in which levels of virus oncogene expression per DNA template varied ~6.6-fold. Across the cells examined, higher levels of virus expression per template were associated with more open chromatin at the HPV16 long control region, together with greater loading of chromatin remodelling enzymes and lower nucleosome occupancy. There were higher levels of histone post-translational modification hallmarks of transcriptionally active chromatin and lower levels of repressive hallmarks. There was greater abundance of the active/elongating form of the RNA polymerase-II enzyme (RNAPII-Ser2P), together with CDK9, the component of positive transcription elongation factor b complex responsible for Ser2 phosphorylation. The changes observed were functionally significant, as cells with higher HPV16 expression per template showed greater sensitivity to depletion and/or inhibition of histone acetyltransferases and CDK9 and less sensitivity to histone deacetylase inhibition. We conclude that virus gene expression per template following HPV16 integration is determined through multiple layers of epigenetic regulation, which are likely to contribute to selection of individual cells during cervical carcinogenesis.

## Introduction

Infection with high-risk human papillomavirus (HRHPV) is responsible for over 600 000 new cancers per annum, including over 500 000 carcinomas of the cervix.^1^ The majority of cervical malignancies are squamous cell carcinomas (SCCs), which arise from a mixed population of HRHPV-infected cells by clonal selection of cells with the greatest competitive growth advantage.^2, 3^ In ~85% of cervical SCCs the selected cells contain HRHPV DNA that is integrated into host chromosomes. In the remaining ~15% of cases the virus genome remains in the extra-chromosomal (episomal) state, as is also seen in the normal virus lifecycle.^4, 5, 6^

In the squamous epithelial lesions that result from productive HRHPV infections, there are ~100 virus episome copies in each basal layer cell.^7, 8^ In the lower cell layers, the necessary expression of the HRHPV early genes E6 and E7 occurs through transcriptional initiation at the virus early promoter (p97 in the case of the major HRHPV, HPV16), while cell maturation is associated with activation of the virus late promoter (p670 for HPV16) and expression of late virus genes. These events are linked to changes in transcription factor binding and altered chromatin structure, based on histone post-translational modifications (PTMs) at nucleosomes associated with the HRHPV genome.^3, 9, 10, 11, 12, 13^

Integration of HRHPV genomes is thought to occur in premalignant squamous intraepithelial lesions (SILs). The probability of integration increases with time^14^ and multiple integration events are thought to be present across the cells of a polyclonal SIL. However, relatively little is known about how particular cells containing integrated HPV gain a growth advantage over other cells with HPV integrated elsewhere in the genome. Notably, the significance of virus transcriptional deregulation in individual integrants during these early events in cervical carcinogenesis is poorly understood. Most studies to date have concerned the end point of the clonal selection process, by focusing on the virus integrants seen in the SCC cells themselves, and have not addressed the dynamic changes that underpin progression from SILs to carcinomas. It is difficult to study such processes by cross-sectional analysis of clinical samples, as the key events that precede clonal selection early in cervical carcinogenesis occur in the basal epithelial cells of low-grade SILs (LSILs),^4, 15^ which would need to be isolated by tissue micro-dissection. A more informative approach has been to study experimental *in vitro* models, including W12.

The W12 system was developed from a polyclonal culture of cervical squamous cells (keratinocytes) naturally infected with HPV16, which were derived by explant culture of a cervical LSIL.^7^ At early passages, these ‘parental' W12 cells are phenotypically and genetically stable. They allow maintenance of HPV16 episomes at ~100 copies per cell and recapitulate an LSIL in three-dimensional organotypic culture. Following long-term culture of W12, however, the cells lose these properties and closely mirror the virus and host events associated with cervical carcinogenesis *in vivo*, with phenotypic progression of the reformed epithelia to high-grade SIL and then SCC.^4^ These events may be associated with deregulation of episome numbers and transcriptional control (e.g. W12 series 4 and W12E cells).^2, 6^ More typically, however, there is a change in the virus physical state from episomal to integrated, due to loss of trans-repressive episomes and emergence of a clonal population containing the HPV16 integration event that confers the greatest growth advantage.^16^ In different W12 series, different integration sites are seen in the selected cells.^16^

We previously used limiting dilution cloning of polyclonal parental W12 cells at early passage, to sample the range of integration events that exists prior to episome clearance and integrant emergence.^14^ The cells were selected under non-competitive conditions, allowing isolation of clones regardless of whether they had a selective advantage in mixed cell populations. By this method, we derived a series of clones from an identical genetic background that differed only by the site of HPV16 integration into the host genome. The large majority of clones showed no evidence of full-length HPV16 concatemerisation^17^ and were therefore so called type I integrants.^2^ Several clones contained multiple copies of the E6/E7 oncogenes, consistent with local DNA rearrangements following integration. At the early passages examined (i.e. prior to clonal evolution events), all clones recapitulated a premalignant SIL phenotype in organotypic tissue culture, with no evidence of invasiveness.^17^

The W12 clones therefore represent a unique system to examine the host and virus factors that determine selection of a particular HPV16 integrant from the range that exists in a typical polyclonal population of premalignant cervical keratinocytes. Across 17 representative clones analysed, levels of HPV16 E6 and E7 transcripts per cell varied by ~6-fold and correlated closely. Only seven of the clones analysed (41%) showed significantly greater expression of HPV16 E6 and E7 than the episome-containing LSIL-like cells from which they were derived, indicating that HPV integration *per se* does not necessarily lead to increased levels of virus oncogenes per cell.^17^ Interestingly, levels of E6/E7 transcript per DNA template across the clones varied by ~16-fold.^17^

In the present study, we used the W12 clones to investigate how different HPV16 integration events in basal-type premalignant cervical keratinocytes lead to different levels of virus oncogene expression. In order to provide a tractable system for our experiments, we chose cells without full-length HPV16 concatemerisation and with four or less copies of integrated virus DNA per cell. Of the five such clones available, two (F and A5) showed high levels of E6/E7 expression per template, two (D2 and H) showed medium levels and one (G2) showed low levels, with ~6.6-fold variation in expression levels across the five clones (Table 1). In our previous preliminary analysis of a restricted sequence of the HPV16 genome in the five clones,^17^ we found that levels of HPV16 expression per template were associated with different distributions of a selected small number of histone PTMs.^17, 18^ We therefore hypothesised that variation in levels of expression per DNA template following HPV16 integration were due to epigenetic differences in the virus chromatin. We used the W12 clones to undertake a detailed and extensive analysis of the epigenetic landscape on the integrated HPV16 genome, focussing on the relationships between virus oncogene expression per template and chromatin accessibility, histone PTMs and activity of RNA polymerase-II (RNAPII).

## Results

### HPV16 oncogene expression per template associates with accessibility of virus chromatin

**Figure 1:**
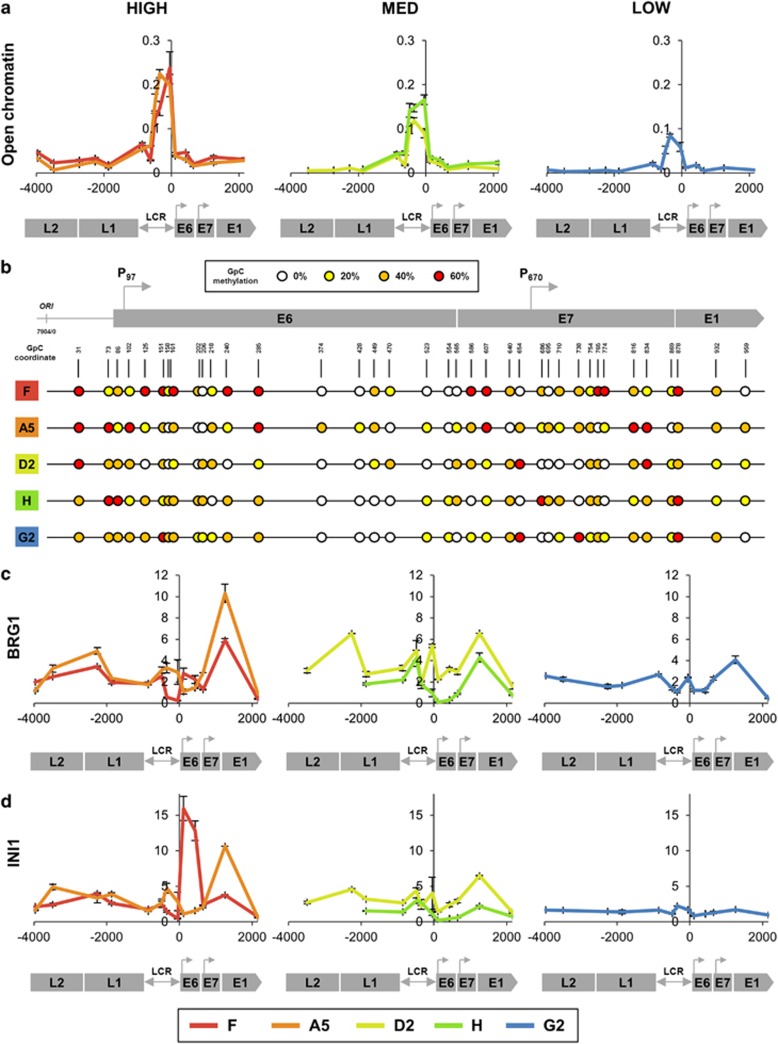
Levels of HPV16 transcription per template associate with virus genome accessibility. (**a**) In each graph the *y*-axis shows fold enrichment of open chromatin across the HPV16 genome, as determined by FAIRE using three biological replicates. Values were normalised to the efficiency of enrichment, as determined by the ratio of *GAPDH* promoter to *GAPDH* open reading frame qPCR. The *x*-axis and underlying schematic show the region of the HPV16 genome analysed. The panels show data for the clones in which transcription levels per template were high, medium (MED) or low. (**b**) Virus genome occupancy by nucleosomes or other DNA-binding proteins, as determined by NOMe sequencing using four biological replicates. Regions with a lower rate of occupancy are indicated by higher levels of exogenously applied GpC methylation. The degree of GpC methylation is shown as a heat map (see key), with circles at individual nucleotide positions. (**c, d**) Association of chromatin remodelling enzymes BRG1 and INI1 with the integrated HPV16 genome across the cell lines. The *y*-axis shows relative levels of enrichment of BRG1 (**c**) and INI1 (**d**), derived from three biological replicates in each case and normalised to host control target regions (see Supplementary Table S1). The *x*-axis and underlying schematic show the region of the HPV16 genome analysed. In all panels, data for each of the five clones are colour coded according to the key at the foot of the figure. This code is maintained in all subsequent figures. In all panels, bars=mean±s.e.m. Abbreviation: FAIRE, formaldehyde-assisted isolation of regulatory element; NOMe, nucleosome occupancy and methylation.

No mutations were seen in any of the five W12 clones following PCR amplification and sequencing of the HPV16 long control region (LCR) (data not shown). By formaldehyde-assisted isolation of regulatory elements, enrichment of open chromatin (i.e. with lower nucleosome occupancy) at the HPV16 LCR and early promoter was greatest in cells with high levels of virus gene expression per template (F and A5) and showed progressive reductions through cells with medium expression per template (D2 and H) to cells with low expression per template (G2) (Figure 1a). The positions of nucleosomes, as indicated by nucleosome occupancy and methylome sequencing, were similar across all clones and usually spaced 150–200 bp apart. These positions were indicated by low levels of exogenously applied GpC methylation. However, clones with high expression per template showed greater amounts of exogenously applied GpC methylation at the early promoter and directly after the transcription start site (Figure 1b), indicating lower average occupancy of the nucleosomes and therefore greater chromatin accessibility in this region. Cells with higher virus expression levels per template also showed a greater abundance of the ATP-dependent chromatin remodelling enzymes BRG1 and INI1 across the virus genome (Figures 1c and d), in keeping with greater openness/accessibility of the HPV16 chromatin in these cells. Both enzymes were most abundant over the virus early region, including the early and late promoters. There was a striking peak of INI1 abundance at the early promoter and transcription start site in clone F.

### High HPV16 expression requires activating chromatin marks

**Figure 2:**
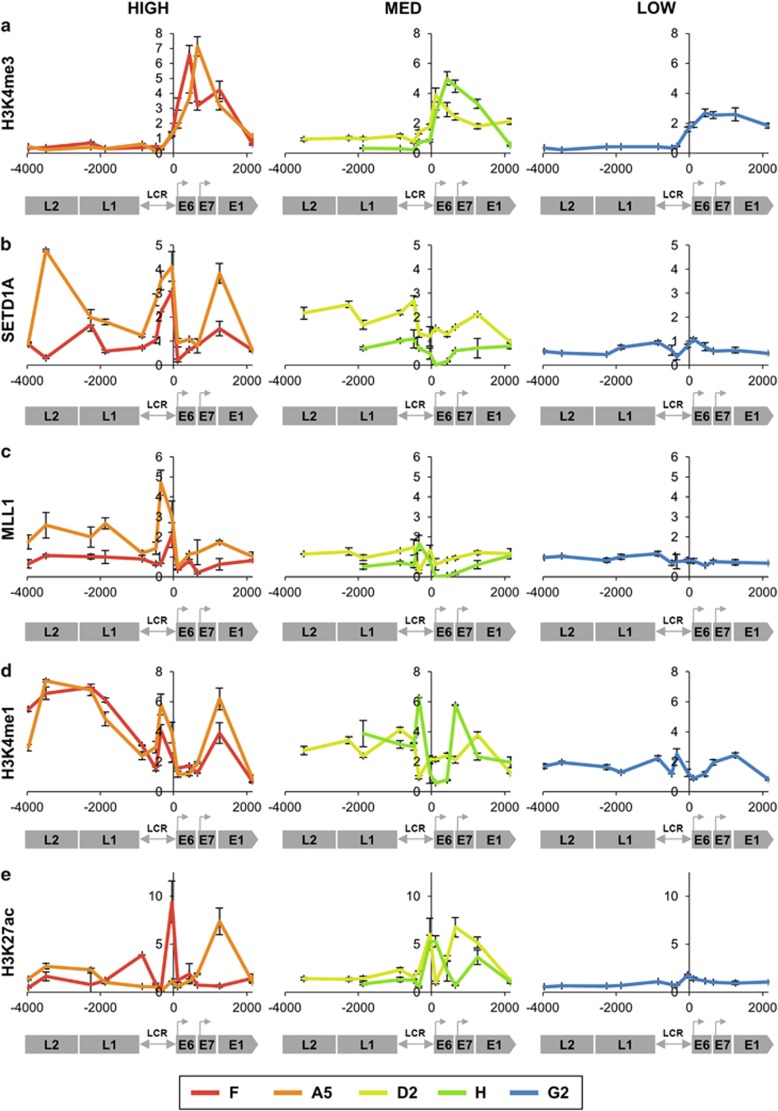
Associations with active histone PTMs and modifying enzymes. Levels of association of the H3K4me3 histone PTM (derived from four biological replicates) (**a**) and the associated histone-modifying enzymes SETD1A (three replicates) (**b**) and MLL1 (four replicates) (**c**); as well as the transcriptional enhancer marks H3K4me1 (three replicates) (**d**) and H3K27ac (two replicates) (**e**). In each graph, the *y*-axis shows the relative levels of enrichment, normalised to host control target regions (see Supplementary Table S1). The *x*-axis and underlying schematic show the region of the HPV16 genome analysed. In all panels, data are colour coded according to the key at the foot of the figure. Bars=mean±s.e.m.

We next quantified levels of histone PTMs on the integrated virus chromatin. Higher virus expression per template was associated with greater levels of histone 3 lysine 4 tri-methylation (H3K4me3), a hallmark of transcriptional activity. We extended our previous observations^17^ by showing that this mark was only present downstream of the HPV16 early promoter and was absent from the late region (Figure 2a). Key enzymatic writers of the mark, SETD1A and MLL1, were also more abundant at the virus genome in cells with higher expression per template, with consistent enrichment at the LCR and early promoter (Figures 2b and c). There was more variable enrichment over the late and early regions, with higher levels of SETD1A in clones A5 and D2 and MLL1 in A5. The cells with high expression per template also showed enrichment of histone PTMs associated with gene enhancer/promoter regions, with strong enrichment of H3K4me1 across the entire virus genome, including the late genes (Figure 2d), and greater abundance of H3K27ac, predominantly at the LCR and early genes (Figure 2e).

**Figure 3:**
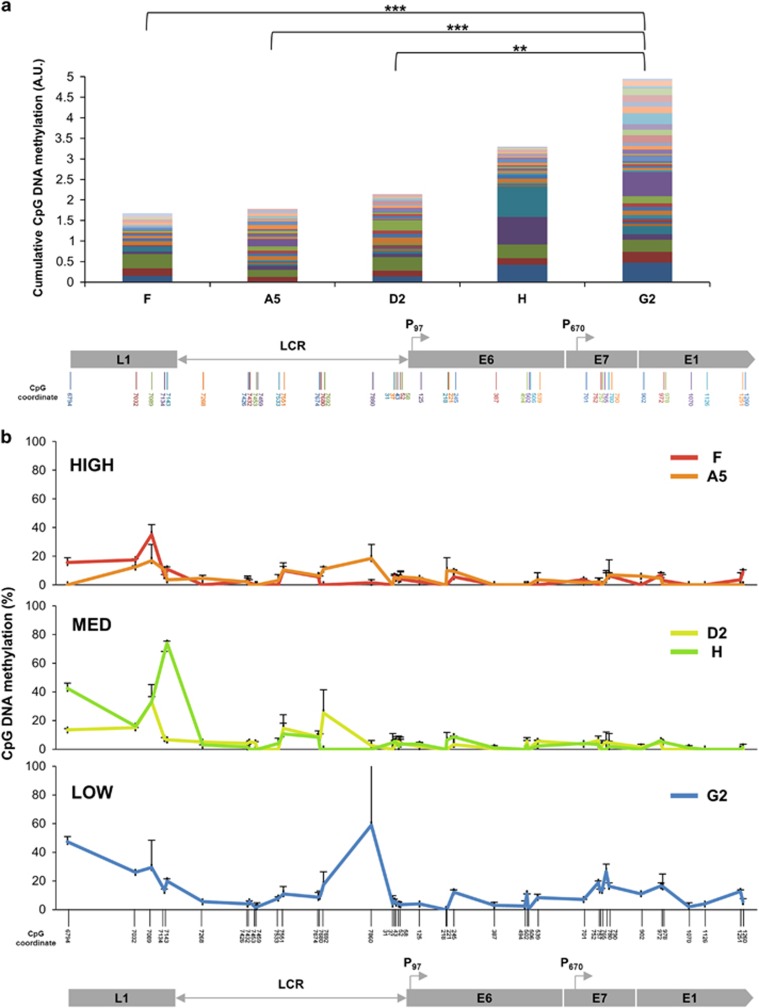
Associations with CpG DNA methylation. (**a**) Cumulative levels of endogenous CpG DNA methylation across the integrated HPV16 genomes, derived from three biological replicates. The coloured bars in each stack correspond to individual CpG sites. The order of the bars in each stack (from bottom to top) corresponds to the order of the CpG coordinates (from left to right) in the genome map at the base of the panel. *P*-values (Student's *t-*test): **P<0.01, ***P<0.001. (**b**) Percentage of endogenous DNA methylation at CpG dinucleotides across the HPV16 genome (*y*-axis). The *x*-axis and underlying schematic show the region of the HPV16 genome analysed. Bars=mean+s.e.m.

Conversely, lower levels of expression per template were associated with higher levels of repressive histone PTMs, namely di-methylation of histone 3 lysines 9 and 27 (H3K9me2 and H3K27me2) (Supplementary Figures S1A and C). However, there was very little enrichment of tri-methylated forms of these histones (H3K9me3 and H3K27me3) at any of the integrated HPV16 genomes (Supplementary Figures S1B and D). The cells with lower expression also showed higher levels of endogenous CpG DNA methylation across the HPV16 genomic region analysed (nt 6731 to 1287) (Figure 3a). There was a prominent peak of DNA methylation at the LCR in G2, the only clone showing low expression per template (Figure 3b). Levels of methylation at *L1* were variable, including between clones with similar levels of virus gene expression per template (Figure 3b).

### HPV16 transcription per template associates with histone methylation modifying enzymes

**Figure 4:**
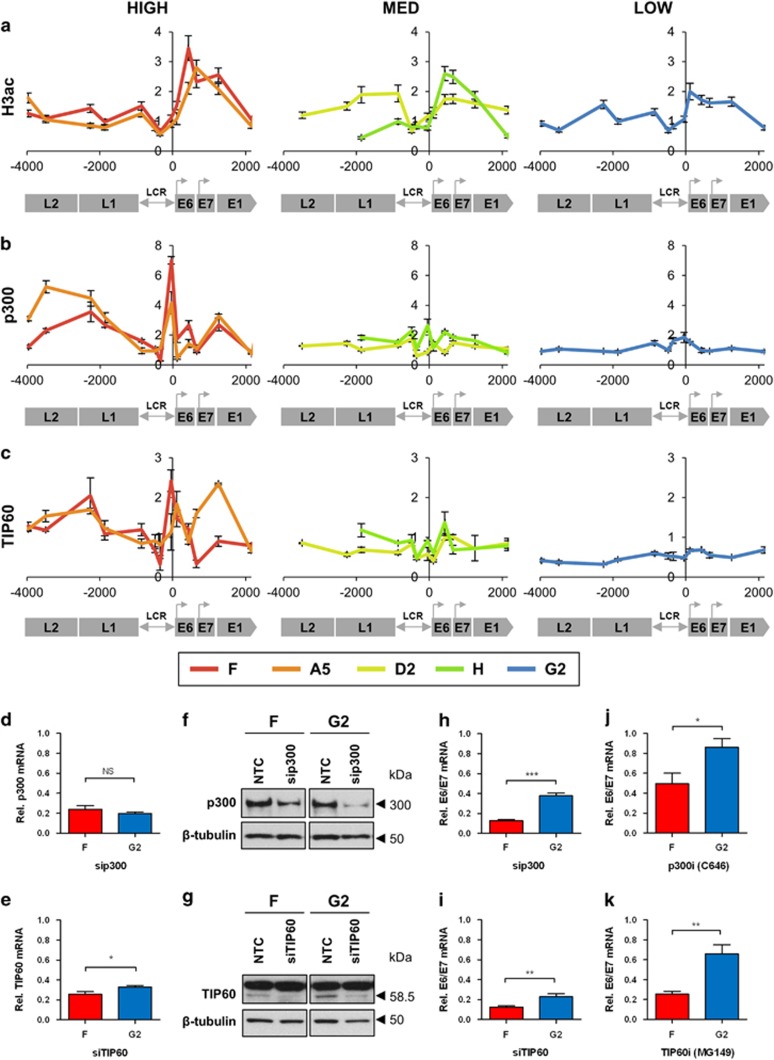
Associations with histone acetylation and HAT abundance/activity. (**a–c**) Levels of the H3ac histone PTM (derived from three biological replicates) (**a**) and the associated HAT enzymes p300 (three replicates) (**b**) and TIP60 (three replicates) (**c**). In each graph, the *y*-axis shows the relative levels of enrichment, normalised to host control target regions (see Supplementary Table S1). The *x*-axis and underlying schematic show the region of the HPV16 genome analysed. In all panels, data are colour coded according to the key beneath panel c. (**d**–**k**) Depletion/inhibition in clones F and G2 of HAT enzymes p300 (upper row) and TIP60 (lower row). The panels show levels of depletion of target mRNAs (**d, e**), target protein (**f, g**) and HPV16 E6/E7 transcripts (**h, i**) in siRNA-treated vs NTC-treated cells, together with HPV16 E6/E7 transcript levels in cells treated with specific small-molecule inhibitors, vs cells treated with vehicle only (**j, k**). All data for p300 were derived from four biological replicates and all data for TIP60 from six biological replicates. Each western blot used protein samples from all replicate experiments combined. Bars=mean±s.e.m. *P-*values (Student's *t-*test): *P<0.05, **P<0.01, ***P<0.001, NS=not significant. Abbreviation: NTC, non-targeting control.

Higher virus expression per template was associated with higher levels of general histone 3 acetylation (H3ac) (Figure 4a), together with greater abundance of the histone acetyltransferases (HATs) p300 and TIP60, across the entire HPV16 genome (Figures 4b and c). The abundance of these enzymes at the HPV16 genome showed no relation to total levels in the cells, indicating specific loading onto the virus chromatin (Supplementary Figure S2). High levels of p300 across the HPV16 genome were associated with high overall abundance of cJun (Supplementary Figure S3A), which can act as a p300 recruiter protein.^19^ However, there was no close association between levels of p300 and cJun at individual sites on the virus genome. Levels of TIP60 were not associated with those of its potential recruiter protein YY1 (Supplementary Figure S3B), but did associate closely with levels of H3K4me1 (Figure 2d).

We tested the functional significance of HAT recruitment in determining levels of HPV16 transcript expression. We inhibited p300 or TIP60 in the cells with the highest and lowest levels of virus early gene expression per template (clones F and G2, respectively) (Figures 4d–k). We did not examine post-transcriptional effects on HPV16 oncoprotein levels in these experiments. We observed significantly greater reductions in E6/E7 transcript levels in clone F vs G2 when p300 was depleted using siRNA (Figure 4h) or specifically inhibited using C646 (Figure 4j) and when TIP60 was depleted using siRNAs (Figure 4i) or specifically inhibited using MG149 (Figure 4k).

**Figure 5:**
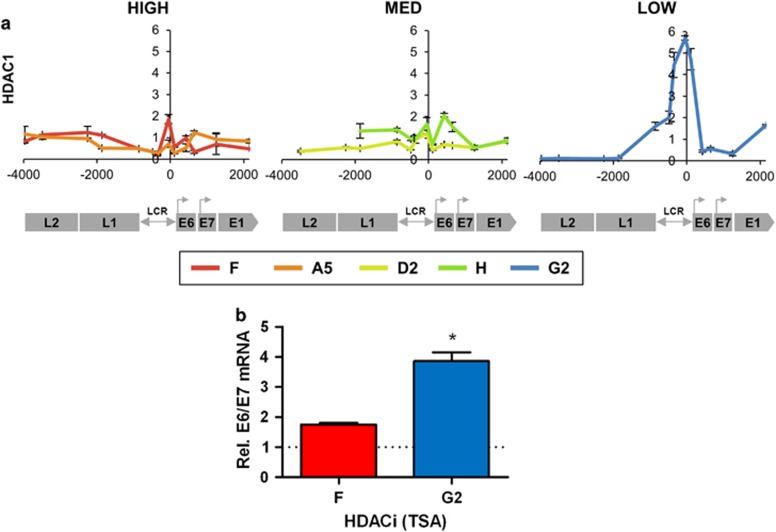
Associations with HDAC abundance/activity. (**a**) Levels of association of HDAC1 enzyme. The *y*-axis shows the relative levels of enrichment, derived from two biological replicates and normalised to host control target regions (see Supplementary Table S1). The *x*-axis and underlying schematic show the region of the HPV16 genome analysed. (**b**) Changes in HPV16 E6/E7 transcript levels following type I/type II HDAC inhibition with TSA in clones F and G2, derived from three biological replicates. Bars=mean±s.e.m. *P-*values (Student's *t-*test): *P<0.05.

Mirroring these observations with HATs, cells with lower virus transcript levels per template showed higher abundance of histone deacetylase 1 (HDAC1) (Figure 5a). In the absence of specific siRNAs targeting HDAC1, we used the class I/II HDAC-specific small-molecule inhibitor Trichostatin-A. After 16 h of treatment, this produced significantly greater increases in HPV16 E6/E7 transcript levels in clone G2 than in clone F (Figure 5b).

### Transcript levels per template associate with active RNAPII, determined by P-TEFb (CDK9)

**Figure 6:**
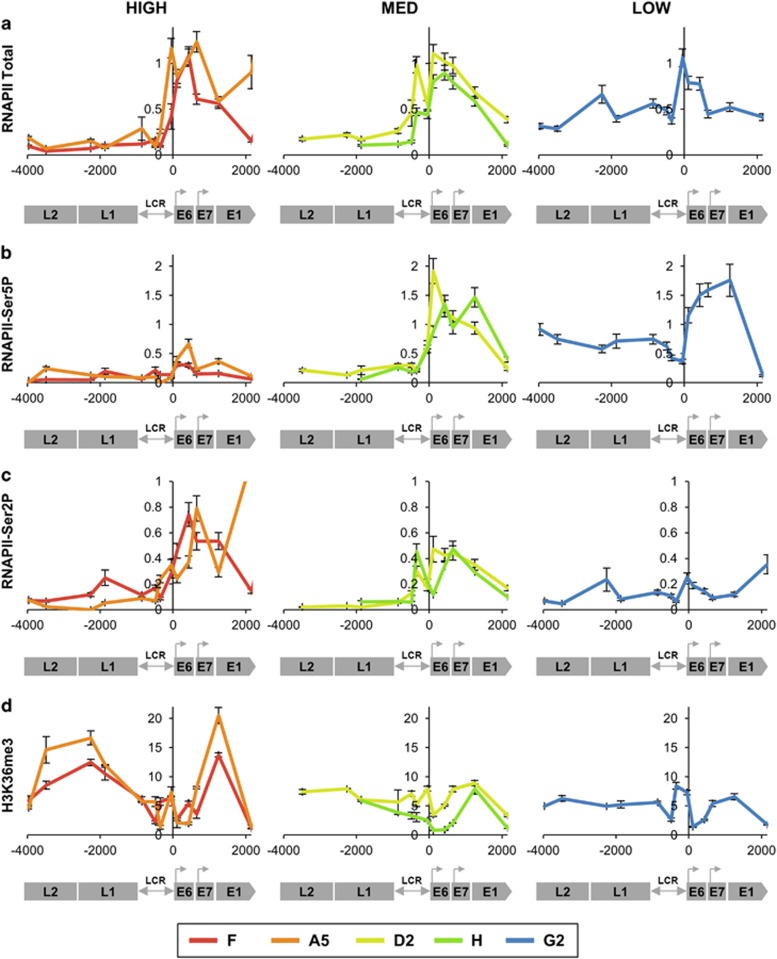
Associations with RNAPII and H3K36me3. Levels of association of total RNAPII (derived from three biological replicates) (**a**), RNAPII-Ser5P (poised/paused) (three replicates) (**b**), RNAPII-Ser2P (active/elongating) (three replicates) (**c**) and H3K36me3 (two replicates) (**d**). In each graph, the *y*-axis shows the relative levels of enrichment, normalised to host control target regions (see Supplementary Table S1). The *x*-axis and underlying schematic show the region of the HPV16 genome analysed. In all panels, data are colour coded according to the key at the foot of the figure. Bars=mean±s.e.m.

**Figure 7:**
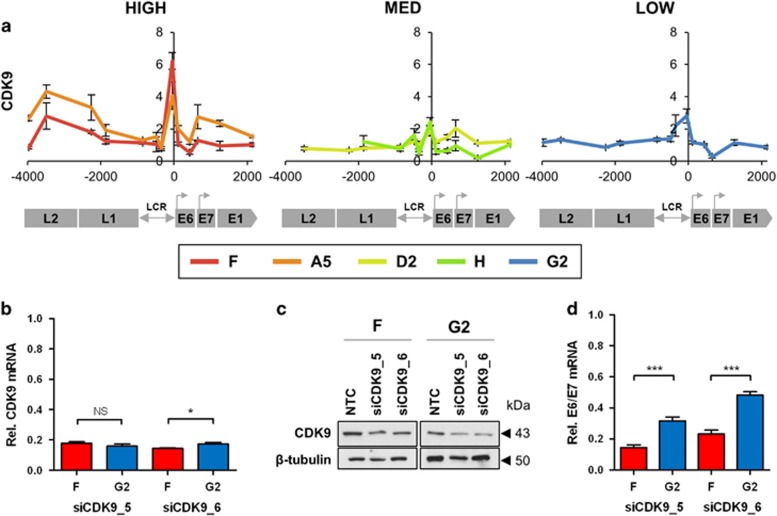
Associations with CDK9 abundance/activity. (**a**) Levels of association of CDK9. The *y*-axis shows the relative levels of enrichment, derived from three biological replicates and normalised to host control target regions (see Supplementary Table S1). The *x*-axis and underlying schematic show the region of the HPV16 genome analysed. (**b–d**) Depletion of CDK9 using siRNAs, showing levels of target mRNA (**b**) and protein (**c**), together with changes in HPV16 E6/E7 transcript levels (**d**), in siRNA-treated vs NTC-treated cells. All data were derived from two biological replicates. The western blot used protein samples from both replicates combined. Bars=mean±s.e.m. *P*-values (Student's *t-*test): *P<0.05, **P<0.01, ***P<0.001, NS=not significant. Abbreviation: NTC, non-targeting control.

There were no differences across the clones in the overall amounts of RNAPII associated with the HPV16 genome (Figure 6a). However, cells with lower virus expression per template showed higher amounts of the poised/paused or stalled form of RNAPII, Ser5P, across the early genes (Figure 6b). Conversely, cells with higher expression per template showed greater amounts of the active/elongating form of RNAPII, Ser2P, across the virus LCR and early genes (Figure 6c), together with higher levels of histone 3 lysine 36 tri-methylation (H3K36me3), a histone PTM associated with transcriptional elongation (Figure 6d). There were also higher levels of the positive transcription elongation factor b (P-TEFb) complex kinase CDK9 (Figure 7a), which is responsible for phosphorylation of the RNAPII C-terminal domain at Ser2. Depletion of CDK9 (Figures 7b and c) produced significantly greater reductions in E6/E7 transcript levels in clone F (higher expression per template) than in clone G2 (lower expression per template) (Figure 7d) (F vs G2 *P*<0.001).

**Figure 8:**
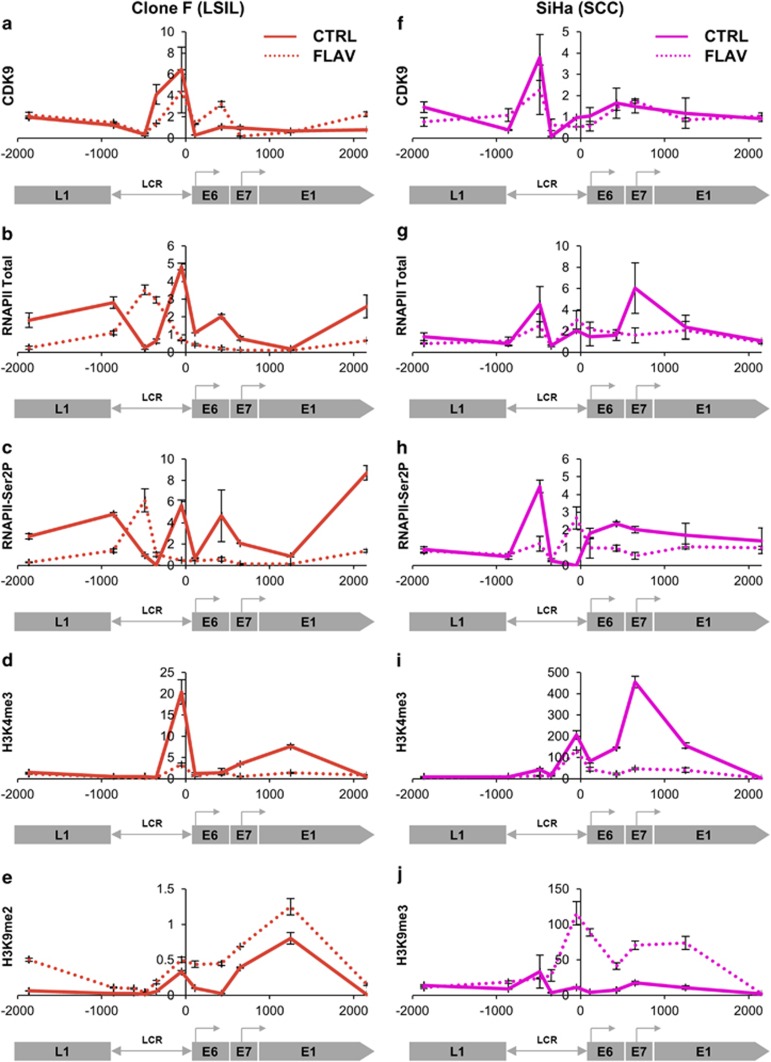
Effects of CDK9 inhibition. Effects of Flavopiridol in clone F (LSIL phenotype) and SiHa (SCC phenotype). Rows show levels of CDK9 (**a, f**), total RNAPII (**b, g**), RNAPII-Ser2P (active/elongating) (**c, h**), H3K4me3 (active) (**d, i**) and H3K9me2/3 (repressed) (**e, j**). In each graph, the *y*-axis shows the relative levels of enrichment, derived from two biological replicates and normalised to host control target regions (see Supplementary Table S1). The *x*-axis and underlying schematic show the region of the HPV16 genome analysed. Solid lines=control-treated cells; dotted lines=Flavopiridol-treated cells. Bars=mean±s.e.m.

We next investigated the consequences of inhibiting CDK9 function in high expressing clone F cells. As the chromatin yield from siRNA experiments was too low, we used the small-molecule inhibitor Flavopiridol, which caused 87% reduction in E6/E7 transcript levels (Supplementary Figure S4A). Similar effects were also seen using other small inhibitors with predominant specificity for CDK9 (Supplementary Figure S4A). While Flavopiridol produced no change in overall levels of CDK9 recruitment at the HPV16 genome (Figure 8a), there were reduced levels of total RNAPII, particularly downstream of the virus early promoter (Figure 8b). There was also less elongating RNAPII-Ser2P downstream of the transcription start site, with evidence of redistribution to the LCR/early promoter region (Figure 8c). In addition, the LCR and early genes showed striking decreases in the histone PTM mark of transcriptional activation, H3K4me3 (Figure 8d), mirrored by increases in the mark of constitutive heterochromatin and transcriptional repression, H3K9me2 (Figure 8e).

Similar observations to those made in clone F were seen in the cervical SCC cell line SiHa. Indeed, the CDK9 inhibitors (including Flavopiridol) produced greater reductions in E6/E7 expression in SiHa than in clone F (95%) (Supplementary Figure S4A), while Flavopiridol led to more pronounced shifts in epigenetic marks and reduced RNAPII-Ser2P levels (Figures 8f–j). Transcript levels in SiHa reduced by >80% over the first 8 h of Flavopiridol treatment (Supplementary Figure S4B), consistent with profound transcriptional shut off of the integrated HPV16 DNA. These changes were associated with complete inhibition of cell growth (Supplementary Figure S4C).

## Discussion

The W12 cell clones represent a unique resource that has enabled us to study the factors associated with the large differences in virus oncogene expression per template observed following natural HPV16 integration events in premalignant basal cervical keratinocytes. We focussed on five clones from the same genetic background, in which HPV16 was integrated at low copy number without full-length virus concatemers. The cells were studied at a very early stage after cloning, when levels of E6/E7 varied by ~6.6-fold but the cells had not shown the effects of HPV16 oncoprotein-driven genomic instability and still recapitulated an LSIL phenotype in organotypic tissue culture. Our findings indicate that levels of HPV16 expression following integration are determined through multiple layers of epigenetic regulation.

Our initial data showed that high virus expression per template was associated with open chromatin at the HPV16 LCR, together with greater loading of chromatin remodelling enzymes and less nucleosome occupancy across the HPV16 early promoter and the oncogenes E6/E7. Together, these changes would be expected to increase template accessibility for the cellular transcriptional machinery, enabling transcriptional activation and RNAPII elongation. The reasons for the relative abundance of BRG1 and INI1 over the virus early region are not certain but may be related to the ability of these enzymes to orchestrate long-range interactions between promoter-enhancer regions.^20, 21^

Levels of HPV16 expression per template were positively associated with higher abundance of histone PTMs that marked transcriptionally active chromatin, together with the cognate writer enzymes. The presence of the transcriptional activation mark H3K4me3 was associated with consistent enrichment for the H3K4 methylases SETD1A and MLL1 at the LCR, where the enzymes would be recruited to the activating RNAPII complex. This observation is paralleled by evidence that a specific isoform of MLL5 (MLL5β) is recruited via a distal AP1 site at the HPV18 LCR and is necessary for virus oncogene expression.^22^ The reasons for the different distributions of H3K4me3 and H4K4me1 are unclear and may be related to the relative distribution or balance of the H3K4 methylases and their cofactors.^23^

Expression levels per template were negatively associated with repressive heterochromatin marks and with overall levels of endogenous CpG DNA methylation. In the type I HPV16 integrants studied here, there was no clear relationship between virus expression per template and *L1* methylation. At present, there is considerable interest in using HRHPV methylation as a clinical diagnostic test, for example to triage cytology samples.^24^ Our data indicate a need for further investigations of the associations between HRHPV *L1* methylation and virus parameters (e.g. physical state, presence or absence of full-length concatemers, levels of early gene expression per template), in order to understand better the potentially complex relationship between *L1* methylation and cervical neoplastic progression.

Interestingly, the repressive heterochromatin marks H3K9me2 and H3K27me2 were present at much greater overall abundance than the equivalent tri-methyl marks H3K9me3 and H3K27me3. Previous work has shown a global reduction in H3K27me3 in HRHPV-infected cells, caused by virus-driven upregulation of H3K27 demethylases KDM6A and KDM6B, and inhibition of the polycomb repressive complex 2, the writer of the H3K27me3 mark.^25, 26^ The absence of these heterochromatic tri-methyl marks is also consistent with chromatin immunoprecipitation sequencing data from the HPV18-positive cervical adenocarcinoma cell line HeLa^27^ and analyses of undifferentiated and differentiated squamous epithelial cells containing HPV31 episomal genomes.^11^ Indeed, for naturally occurring HRHPV integrants (as opposed to those generated experimentally) significant levels of heterochromatic marks, including H4K20me3, have only been reported in CaSki cervical SCC cells, in which there is an unusually high number of integrated HPV16 genomes (~600 copies).^28^

Virus expression per template was associated with histone acetylation at the integrated HPV16 genomes, consistent with our previous findings in episome-associated cervical carcinogenesis^6^ and with observations using genetically modified HPV16 templates.^29^ Histone acetylation associated positively with levels of both HATs examined, p300 and TIP60. High levels of p300 were associated with greater overall abundance of cJun, a potential component of the AP1 complex, which is a possible mechanism of p300 recruitment. While it has previously been shown that p300 can activate HPV gene expression,^19, 30, 31^ our data demonstrate a functional, dose-dependent relationship between levels of p300 and HPV16 gene expression, as cells with high virus expression per template showed significantly greater sensitivity to p300 depletion or inhibition than those with low expression per template. Similar observations were made when inhibiting TIP60. Interestingly, there is evidence that TIP60 is a transcriptional repressor at the HPV18 early enhancer/promoter and can be targeted for degradation by the HPV18 E6 protein.^31, 32^ Inhibition using MG149 also indicated an activating role for TIP60 in episome-containing parental W12 cells (data not shown), despite the presence of E2 protein, which has been shown to organise TIP60-mediated repression of the HPV18 LCR.^33^ Therefore, the function of TIP60 at HPV16 genomes is not obviously dictated by template structure.

The reasons for the disparate observations concerning TIP60 function are unclear. The mechanism of TIP60 recruitment may be relevant, as we observed no overall association between levels of TIP60 and YY1 in W12 cells, whereas YY1 was found at the integrated HPV18 genome in HeLa cells, where TIP60 is repressive.^31^ In the absence of YY1, TIP60 can be recruited to chromatin via activated RNAPII-Ser2P itself^34^ and by various other transcription factors including E2F1, MYC, MAX and MXI1, all of which have been found at the HPV18 LCR.^27^ Indeed, increased TIP60 recruitment to the hTERT promoter, likely through MYC interaction, was seen in human foreskin keratinocytes expressing HPV16 E6 protein.^35^ TIP60 has also been shown to interact directly with chromatin through its chromodomain. This can occur via the repressive mark H3K9me3 at DNA double-strand breaks^36^ but also via the active marks H3K4me3 (enabling TIP60 to act as a histone code reader/translator)^37^ and H3K4me1.^38^ The latter, when combined with H3K27ac, is an indicator of active enhancers.^39^ In the W12 cells with high virus expression per template, these marks were present, together with p300, at the integrated HPV16 LCR, which therefore appears to be acting as a canonical enhancer of transcription. Interestingly, such marks were also present over the virus late gene region, which, when out of the context of the episomal genome, may augment integrated HPV16 gene expression.

The differences in HAT recruitment between the clones were mirrored by differences in HDAC1 abundance at the HPV16 genome. HDAC1 levels were greater in cells with less virus gene expression per template, which showed significantly greater increases in transcript levels following HDAC inhibition. However, HDAC1 was detectable at the virus genome in all clones and all showed increased gene expression levels following HDAC inhibition with TSA over a relatively long duration of 16 h. These observations are consistent with data describing the necessity for HDAC presence at gene promoter regions, in order to allow resetting of histone acetylation during the dynamic turnover of these marks that accompanies RNAPII progression.^34, 40^

While virus expression per template showed no association with overall levels of RNAPII at the HPV16 genome, there was an association with levels of the active/elongating form of the enzyme (RNAPII-Ser2P), together with those of CDK9, the component of P-TEFb responsible for phosphorylating Ser2 of the RNAPII C-terminal domain. The CDK9 enzyme was functionally significant, as evidenced by a greater sensitivity to depletion in cells with higher HPV16 gene expression per template. We also observed striking changes in the distribution of RNAPII and chromatin marks following treatment with Flavopiridol. While this small molecule can inhibit multiple CDKs and affect cell cycle progression, its major mode of action is considered to be inhibition of CDK9.^41, 42^ The importance of P-TEFb/CDK9 in transcription of integrated HPV16 supports observations for other viruses. For example, CDK9 is necessary to relieve RNAPII pausing at the Epstein-Barr virus C promoter and drive transcription of polycistronic virus mRNAs,^43^ while P-TEFb is required for Tat-driven transcriptional elongation at the human immunodeficiency virus (HIV) long terminal repeat.^44^

**Figure 9:**
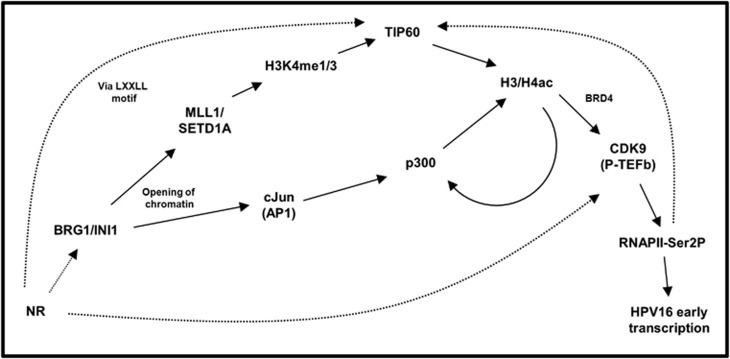
Working model of the multilayered epigenetic changes that enable high levels of virus gene expression per template following HPV16 integration. Recruitment of chromatin remodelling enzymes (BRG1/INI1) to the HPV16 genome, possibly through host steroid hormone nuclear receptors (NR), allows greater accessibility to the virus chromatin of transcription factors (e.g. cJun/AP1) and histone-modifying enzymes, including MLL1 and SETD1A, which can write the H3K4me1/3 marks. Recruitment of HATs can occur though interactions with transcription factors (e.g. p300) and histone PTMs, which may allow TIP60 recruitment through its chromodomain. Once acetylation of histones has occurred, recruitment of CDK9 (the enzymatic component of P-TEFb) is able to activate RNAPII through phosphorylation of Ser2 at the C-terminal domain, leading to stimulation of transcription from the HPV16 early promoter.

Together, our data are consistent with the model shown in Figure 9. Integrated HPV16 templates showing higher levels of oncogene expression are associated with more accessible DNA, via the action of chromatin remodellers. This accessibility leads to the recruitment of activating histone-modifying enzymes, either directly or via transcription factors. In turn, these enzymes methylate and acetylate histone tails, so that the recruitment and activation of RNAPII can occur through activating complexes such as P-TEFb. While the integrated templates with lower expression levels are still able to activate RNAPII, there is a shift in the balance of activating and repressing enzymes that affects gene expression levels. In future work, it will be important to study the mechanisms by which initial virus template accessibility is determined, including whether HPV16 acquires the features of the host chromatin at integration sites. The W12 system will allow detailed dissection of the relative roles of virus factors, such as those described here, and host genes in providing individual cells with a selective advantage during the early stages of cervical neoplastic progression.

## Materials and methods

### Cell culture

Previous publications have given detailed descriptions of the W12 system,^6, 16, 45^ including generation of the W12 cell clones.^14, 17^ The five clones selected for further investigation (Table 1) were episome-free and did not express the HPV16 transcriptional regulator E2.^17^ All W12 cells were grown in monolayer culture, as described,^46^ in order to restrict cell differentiation and maintain the phenotype of the basal epithelial cell layer, the key site of HRHPV transcriptional deregulation in cervical carcinogenesis.^4, 15^ Cells were analysed at the lowest available passage (p) after cloning (typically p3 to p8), in order to minimise any effects of genomic instability caused by deregulated HPV16 oncogene expression. We also used the HPV16-positive cervical SCC cell line SiHa,^47^ which contains ~2 integrated virus copies and was grown as described.^48^

### Treatment with small-molecule inhibitors

Cells were treated for 16 h with medium supplemented with small-molecule inhibitors, using the highest doses that did not produce cell death over the timecourse of the experiments. The small-molecule inhibitors used were: p300 inhibitor, C646 (SML0002; Sigma-Aldrich, Dorset, UK; 25 μm); TIP60 inhibitor, MG149 (Axon 1785; Axon Medchem, Groningen, Netherlands; 150 μm); HDAC inhibitor, Trichostatin-A (T1952; Sigma-Aldrich; 400 nm); or CDK9 inhibitors, Flavopiridol (F3055; Sigma-Aldrich; 150 nm), Roscovitine (C3249; Sigma-Aldrich; 20 μm) or DRB (D1916; Sigma-Aldrich; 50 μm). For analysis of cell growth, cells were seeded at 5 × 10^4^ per well and treated with Flavopiridol after 24 h. Total live cell counts were carried out every 24 h over 5 days, using Trypan blue staining. In all experiments, negative control cells were treated with equivalent volumes of DMSO vehicle (vol/vol).

### Gene depletion

Each target gene was depleted using human Flexitube siRNAs (Qiagen, Crawley, UK): *CDK9* (CDK9_5 SI00605066; CDK9_6 SI00605073); *p300* (EP300_7 SI02626267); *TIP60* (KAT5_2 SI05120304); non-targeting control (AllStars Negative Control siRNA, 1027280). All siRNAs were used at 10 nm, with cells being transfected at 20–30% confluence using Lipofectamine RNAiMAX (Invitrogen, Paisley, UK) as described.^49, 50^

### Quantification of host proteins and HPV16 transcripts

Quantitative western immunoblotting was carried out as described,^6, 17, 51^ using the primary antibodies listed in Supplementary Table S1. Protein concentrations were compared with those of the β-tubulin loading control (Abcam, Cambridge, UK; 6ng/ml), using ImageJ software. Levels of HPV16 E6 and E7 transcripts were measured using SYBRGreen quantitative reverse transcription–PCR (qRT–PCR), as described.^17^ Primers and conditions are given in Supplementary Table S2. Relative transcript levels were determined using the Pfaffl equation,^52^ normalised to the mean of four housekeeping genes^53^ and residual levels of the target protein, then referenced to control samples.

### Chromatin immunoprecipitation

Chromatin immunoprecipitation was performed as described,^6, 17^ using chromatin immunoprecipitation-validated primary antibodies and appropriate serum/IgG negative controls (Supplementary Table S1). In contrast to our previous assessment of a relatively limited region of the HPV16 genome, we analysed 6094 nucleotides (nt) of HPV sequence, from the *L2* gene, through the LCR, to the *E1* gene (nt 3936 to 2158). This genomic region was present in all five clones, with the exception of nt 3936 to 6039 in clone H and nt 3936 to 4419 in clone D2. Primers and conditions used for qPCR are given in Supplementary Table S3. Efficiency of immunoprecipitation of each target was normalised using control region qPCR primers (Supplementary Table S4).

### Formaldehyde-assisted isolation of regulatory elements and nucleosome occupancy and methylome sequencing

Formaldehyde-assisted isolation of regulatory element was carried out as described.^54^ Quantification of HPV16 DNA sequences was carried out by qPCR and normalised to the efficiency of enrichment, as determined by the ratio of *GAPDH* promoter (open)^55^ to *GAPDH* open reading frame (closed).^43^ Primers and conditions for qPCR were those in Supplementary Tables S3 and S4. The occupancy of nucleosomes or other DNA-binding proteins between the HPV16 early promoter and *E1* gene (nt 7902 to 1012) was assessed by nucleosome occupancy and methylome sequencing (Active Motif, La Hulpe, Belgium), which measures the distribution of exogenous GpC DNA methylation.^56^ Samples were amplified in duplicate using PCR primers designed to exclude either GpC or CpG dinucleotides, in order to eliminate amplification bias (Supplementary Table S5). PCR products were Sanger sequenced, using 5′- and 3′-end primers to confirm reads from each end of the product. Each analysis was carried out in duplicate and the degree of cytosine methylation for each nucleotide position averaged across replicates. Percentage GpC methylation was scored in 20% intervals, from which a heatmap was generated.

### HPV16 DNA methylation

Five hundred nanograms of genomic DNA were bisulphite-converted using the EpiTect Bisulfite Kit (59104; Qiagen), then desulphonated, washed and eluted in 40 μl of buffer. PCR amplification of HPV16 sequences was carried out using Immolase (Bioline, London, UK) and the primers listed in Supplementary Table S6. *LINE1* amplification was also carried out as a methylation-positive conversion control. Sequencing primers were designed using PyroQ software (Pyromark MD, Qiagen) and analysis performed on a Pyromark MD pyrosequencer, using standard protocols and controls. For each cell line, assays were performed in duplicate on a minimum of three independently prepared bisulphite-converted DNA samples.

## Figures and Tables

**Table 1:** Details of the W12 clones studied

*Clone*	*Integration site*	*Ploidy*	*HPV16 gene copy number*					*HPV16 E6/E7 expression per template*	*Expression per template category*
			*E6*	*E7*	*Mean E6/E7*	*E2-5′*	*E2-3′*		
F	4q13.3	2N	1	1	1	1	1	248.6 (±31.8)	HIGH
A5	8p11.21	2N	1	1	1	1	1	215.6 (±14.9)	
D2	18q21.2	2N	3	4	4	0	3	118.5 (±12.0)	MEDIUM
H	4q21.23	2N	1	1	1	0	1	100.1 (±12.4)	
G2	21q22.1	2N	3	3	3	3	0	37.5 (±4.2)	LOW

All virus gene copy numbers were adjusted for cell ploidy and rounded to the nearest whole number. Levels of HPV16 E6 and E7 transcripts per template were referenced individually to low passage episome-containing W12 cells (W12 Series6 p11) and mean values (±s.e.m.) were determined from three biological replicates. Clone G2 showed three different virus–host junction transcripts by RNA-sequencing and clone D2 showed four different virus–host junction transcripts (data not shown). All clones tested (F, A5, D2 and G2) reformed an LSIL in organotypic tissue culture.
