# Supplementary material for: HPV16 oncogene expression levels during early cervical carcinogenesis are determined by the balance of epigenetic chromatin modifications at the integrated virus genome
Source: Oncogene. 2016 Feb 15;35(36):4773–86. doi: 10.1038/onc.2016.8 (PMC5024154; doi:10.1038/onc.2016.8)
Supplement: Supplementary Information [file onc20168x1.docx]

**Supplementary Information**

**Legends to Supplementary Figures**

**Supplementary Figure-S1. Correlations with repressive histone PTMs.** Levels of association of: H3K9me2 (derived from three biological replicates) (A), H3K9me3 (three replicates) (B), H3K27me2 (three replicates) (C) and H3K27me3 (four replicates) (D). In each graph, the y-axis shows the relative levels of enrichment, normalised to host control target regions (see Supplementary Table-S1). The x-axis and underlying schematic show the region of the HPV16 genome analysed. In all panels, data are colour coded according to the key at the foot of the figure. Bars = mean ±SEM.

**Supplementary Figure-S2. Overall levels per cell of enzymes detected by ChIP.** Baseline levels were determined by Western blot (A-D) and quantified using Image J (E-H). The enzymes assessed were: p300 (one biological replicate) (A, E), TIP60 (two replicates) (B, F), HDAC1 (one replicate) (C, G) and CDK9 (two replicates) (D, H). For each blot, levels of the target and loading control were quantified at two or three different exposures and the mean values determined. Where more than one blot was performed, a representative image is shown. Bars = mean ±SEM. *P* values (Student’s T test): **P*<0.05, ***P*<0.01, ****P*<0.001. A.U. = arbitrary units.

**Supplementary Figure-S3. Correlations with HAT-recruiting transcription factors.** Levels of association of the HAT-recruiting transcription factors: cJun (A) and YY1 (B). In each graph, the y-axis shows the relative levels of enrichment, derived from two biological replicates in each case and normalised to host control target regions (see Supplementary Table-S1). The x-axis and underlying schematic show the region of the HPV16 genome analysed. In both panels, data are colour coded according to the key at the foot of the figure. Bars = mean ±SEM.

**Supplementary Figure-S4. Further effects of CDK9 inhibition.** (A) Levels of depletion of HPV16 E6/E7 transcripts in clone F (red) and SiHa (pink) cells treated for 16 hours with Flavopiridol, Roscovitine or DRB, vs. cells treated with vehicle only. Data were derived from three biological replicates in each case. (B) Timecourse of HPV16 E6/E7 transcript depletion in SiHa cells treated with Flavopiridol, vs. cells treated with vehicle only. Data were derived from three biological replicates. (C) Growth of SiHa cells treated with Flavopiridol, vs. cells treated with vehicle only, derived from three biological replicates. Bars = mean ±SEM. *P* values (Student’s T test): ***P*<0.01, ****P*<0.001.
