# Supplementary figures and images for: HPV16 oncogene expression levels during early cervical carcinogenesis are determined by the balance of epigenetic chromatin modifications at the integrated virus genome
Source: Oncogene. 2016 Feb 15;35(36):4773–86. doi: 10.1038/onc.2016.8 (PMC5024154; doi:10.1038/onc.2016.8)

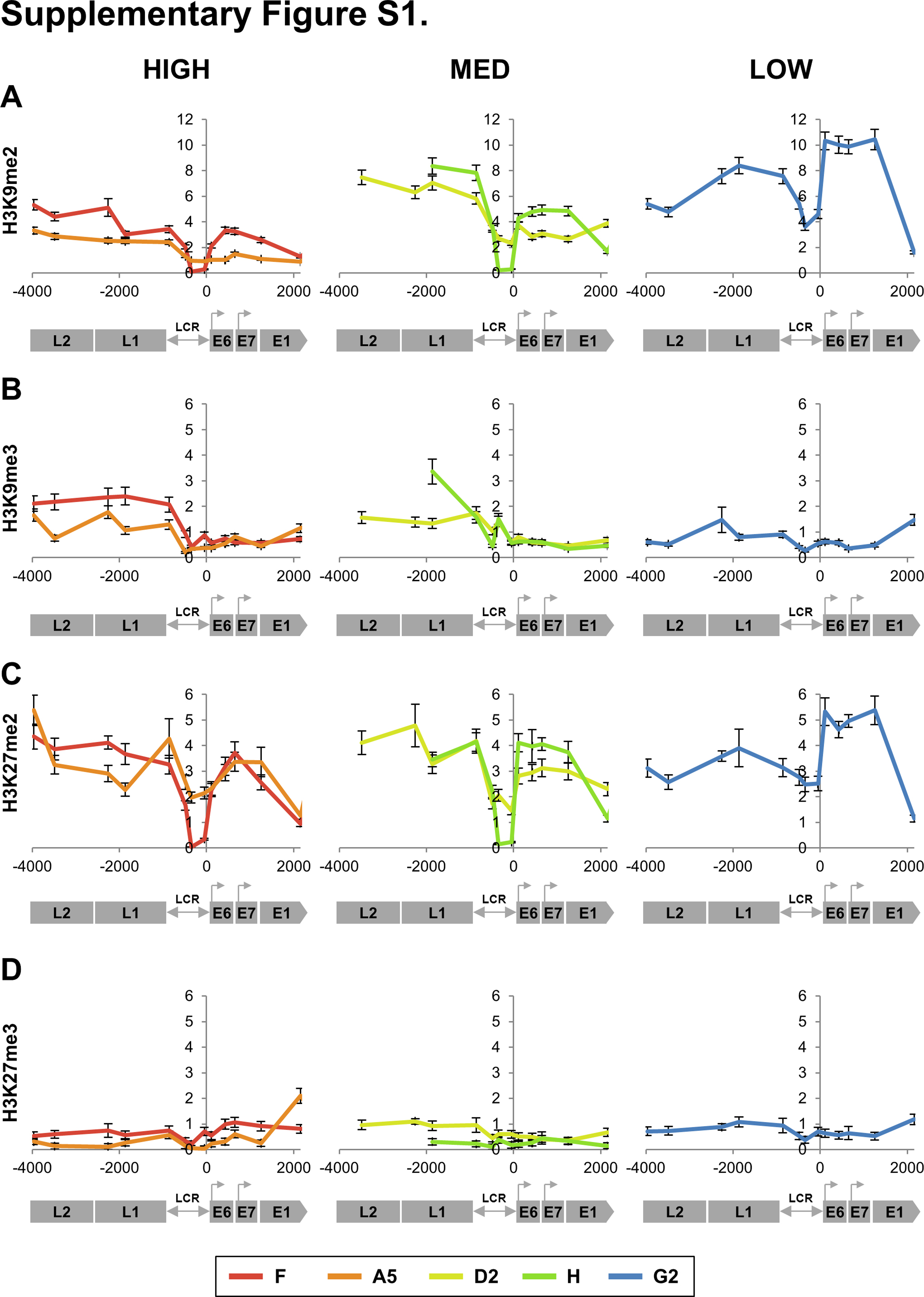

Supplement: Supplementary Figure 1 [file onc20168x2.tif]

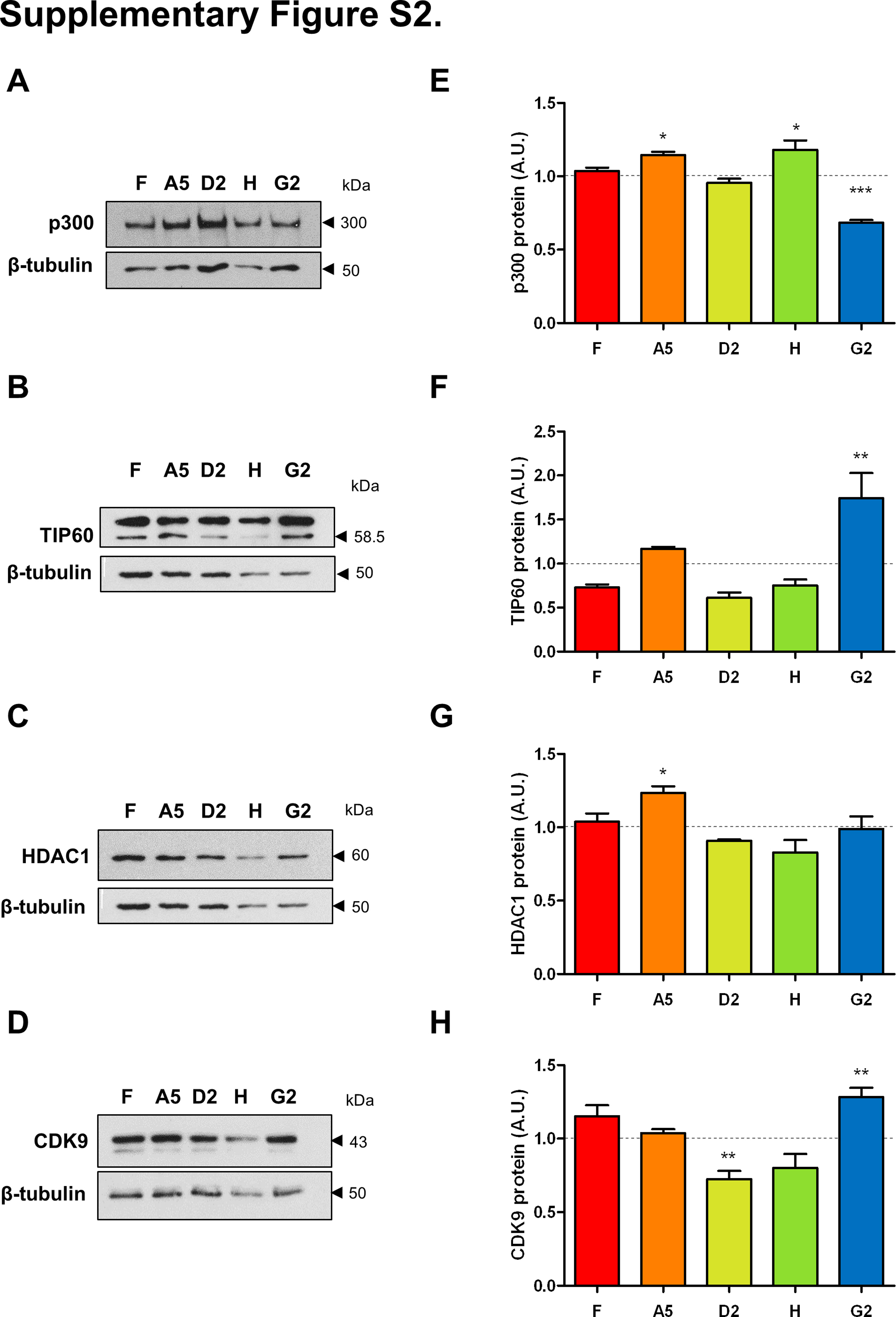

Supplement: Supplementary Figure 2 [file onc20168x3.tif]

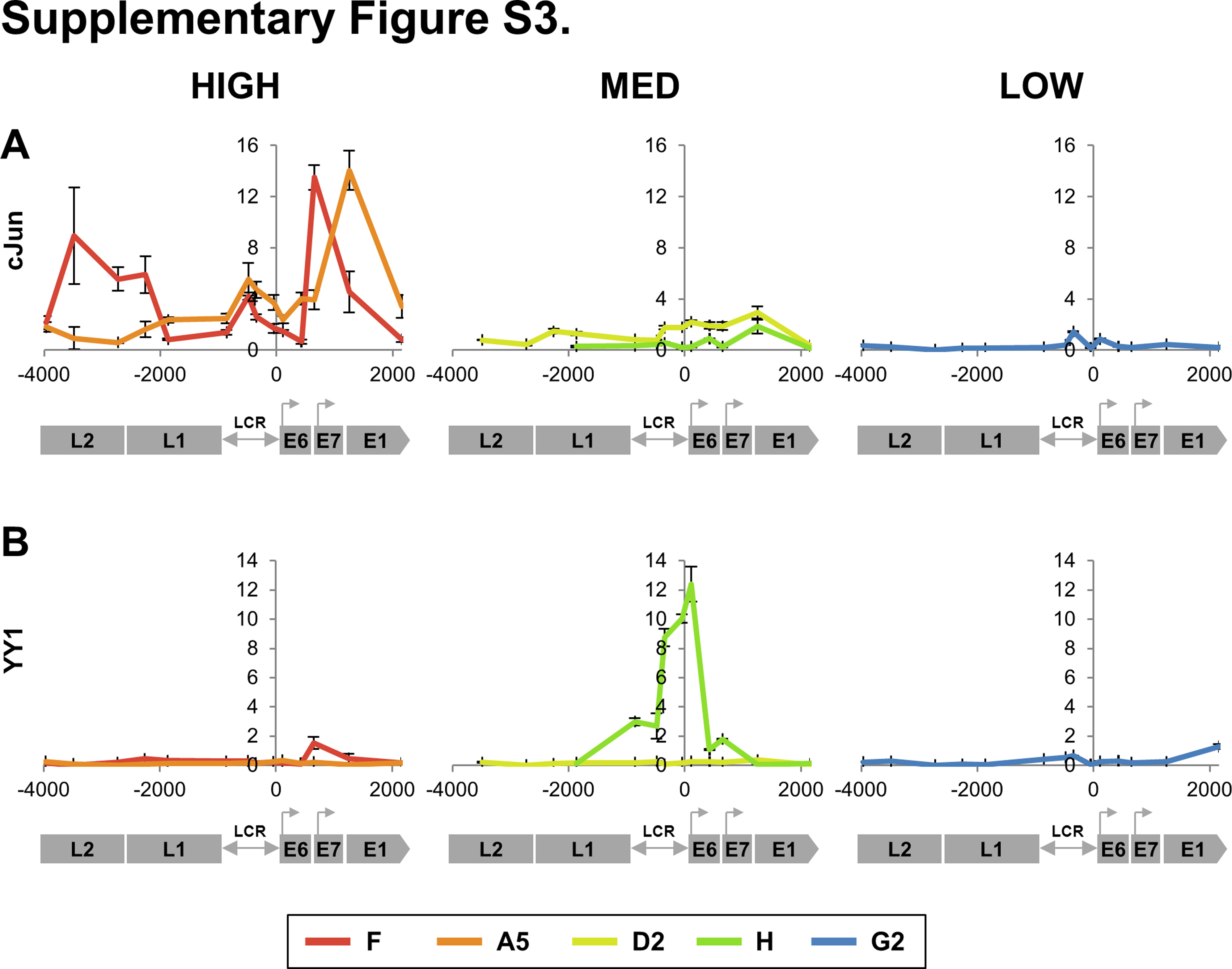

Supplement: Supplementary Figure 3 [file onc20168x4.tif]

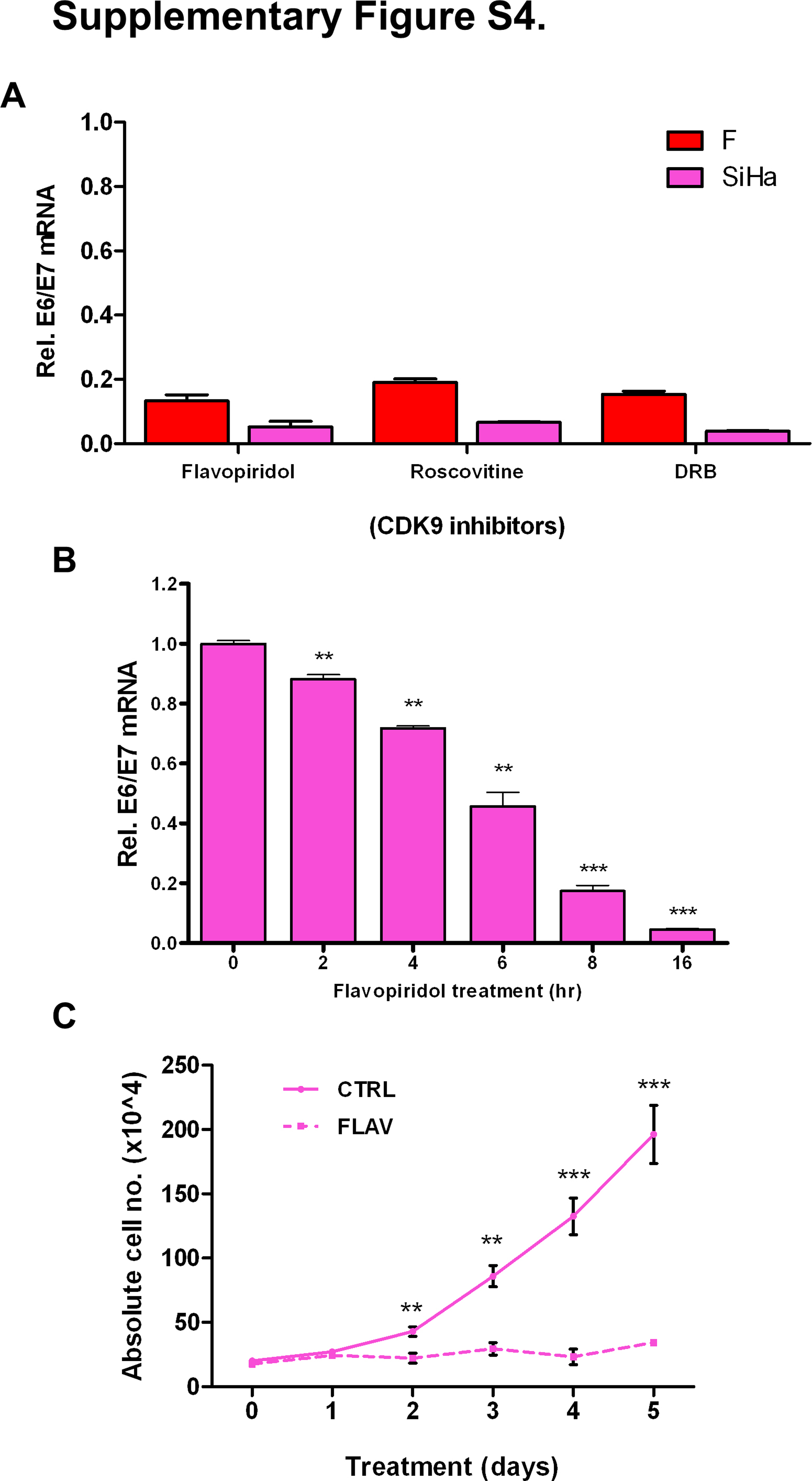

Supplement: Supplementary Figure 4 [file onc20168x5.tif]

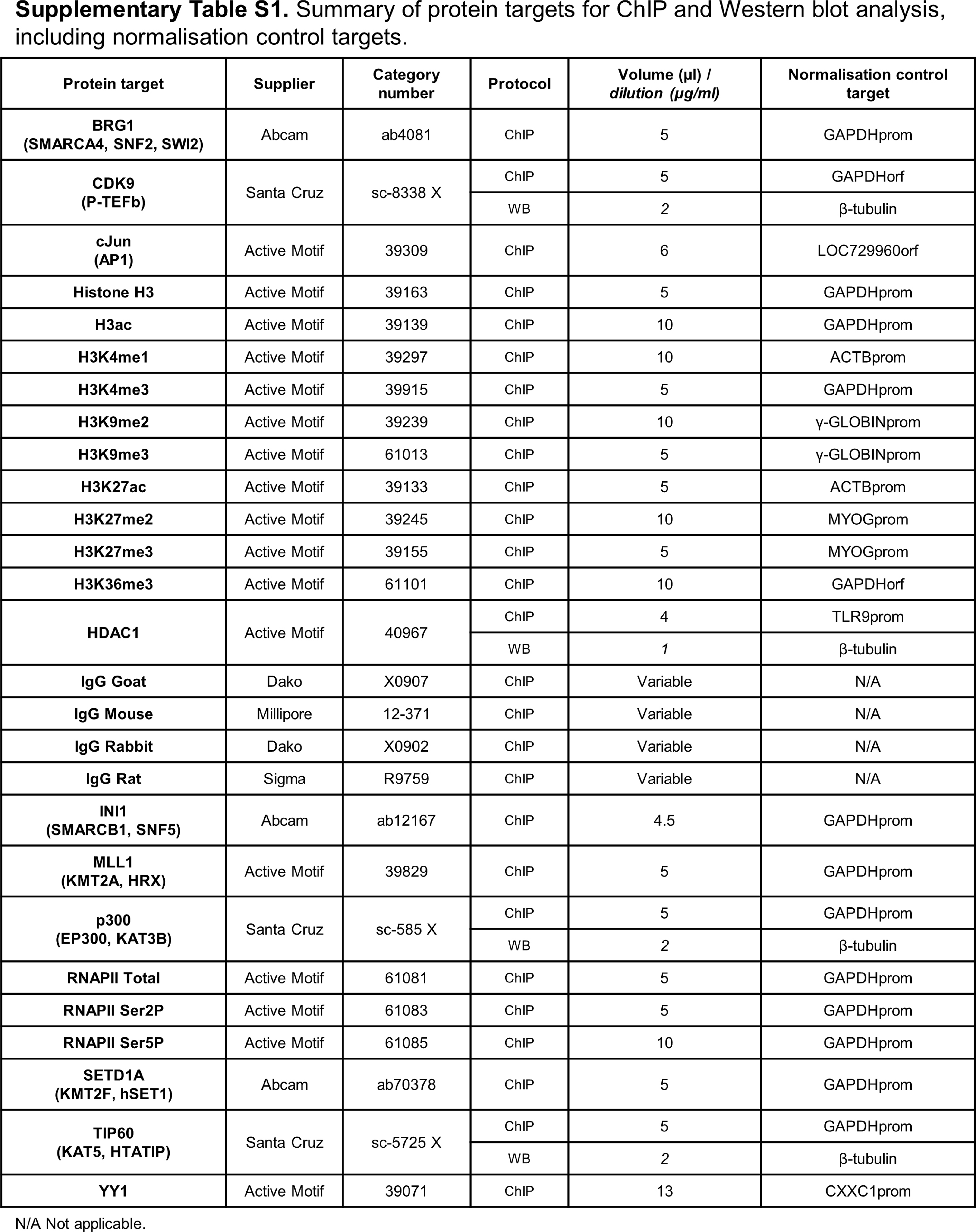

Supplement: Supplementary Table 1 [file onc20168x6.tif]

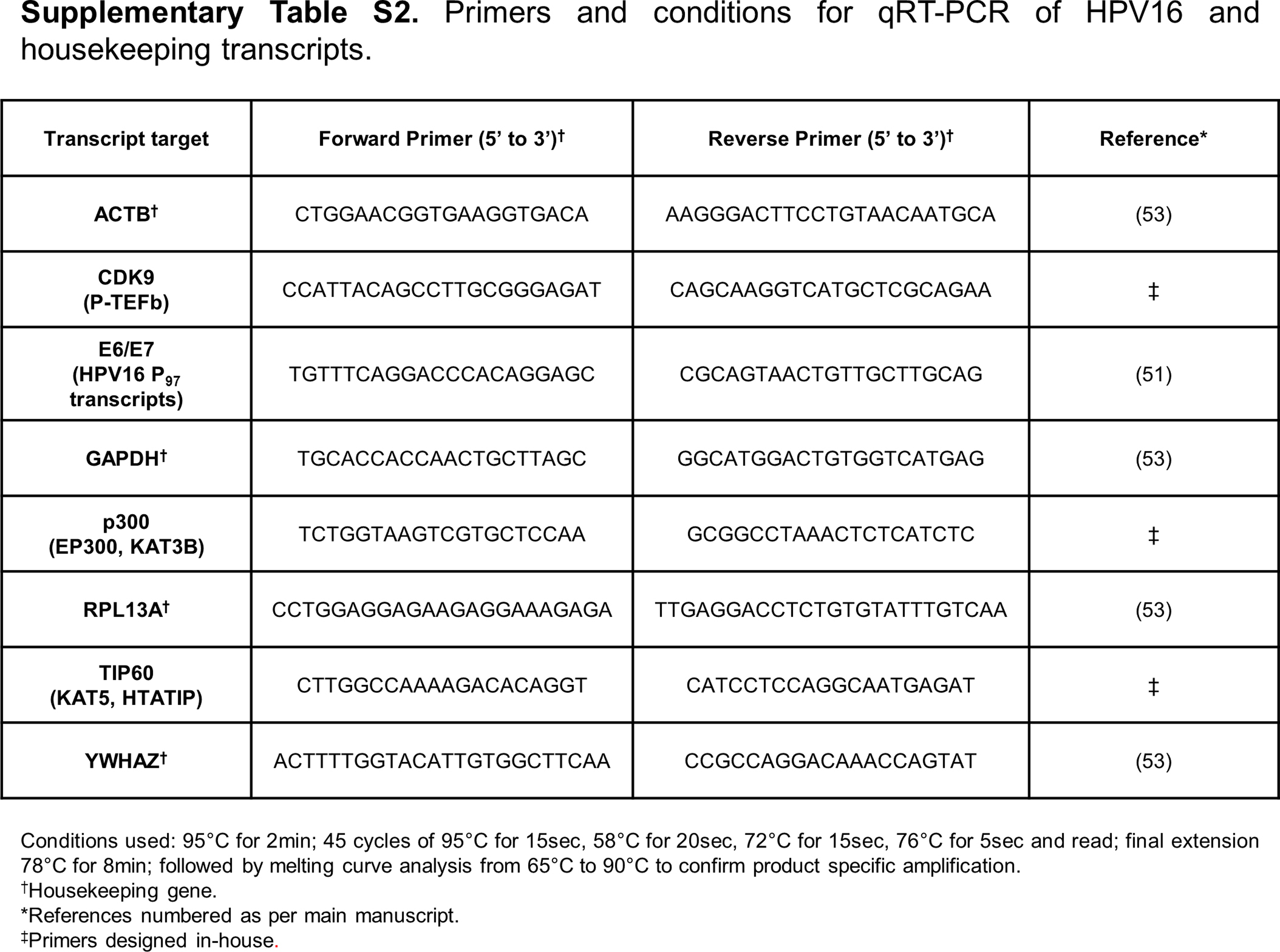

Supplement: Supplementary Table 2 [file onc20168x7.tif]

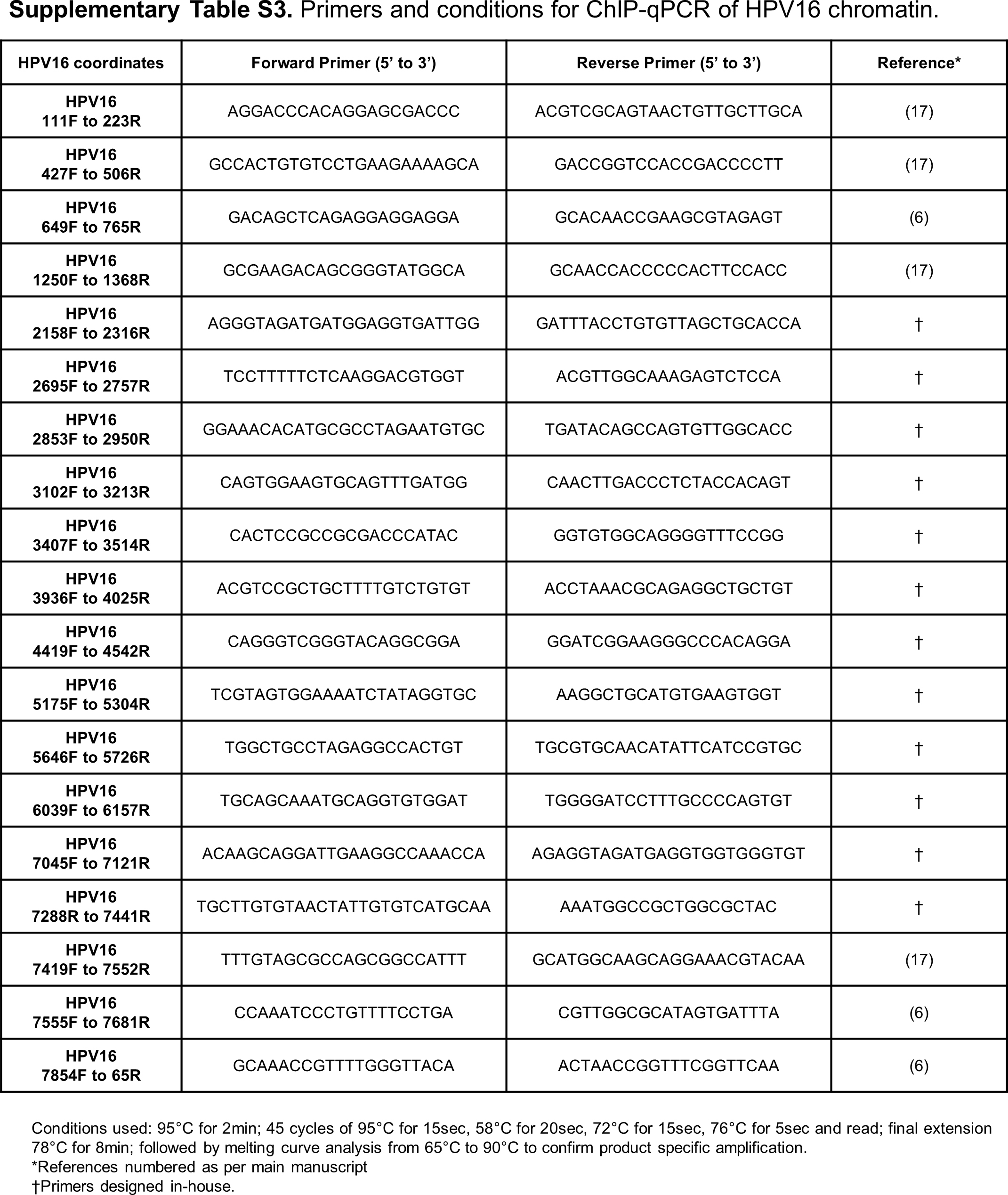

Supplement: Supplementary Table 3 [file onc20168x8.tif]

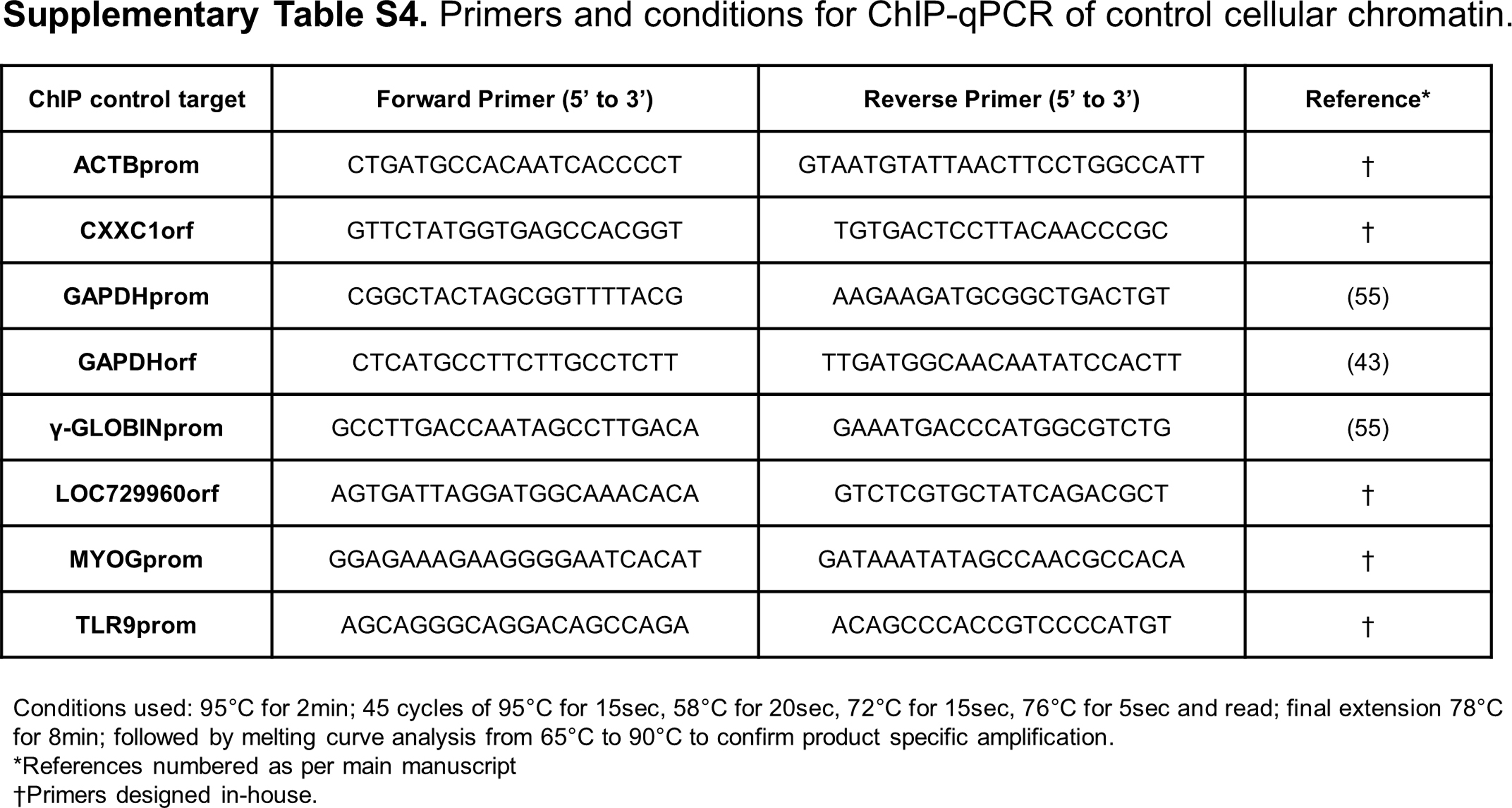

Supplement: Supplementary Table 4 [file onc20168x9.tif]

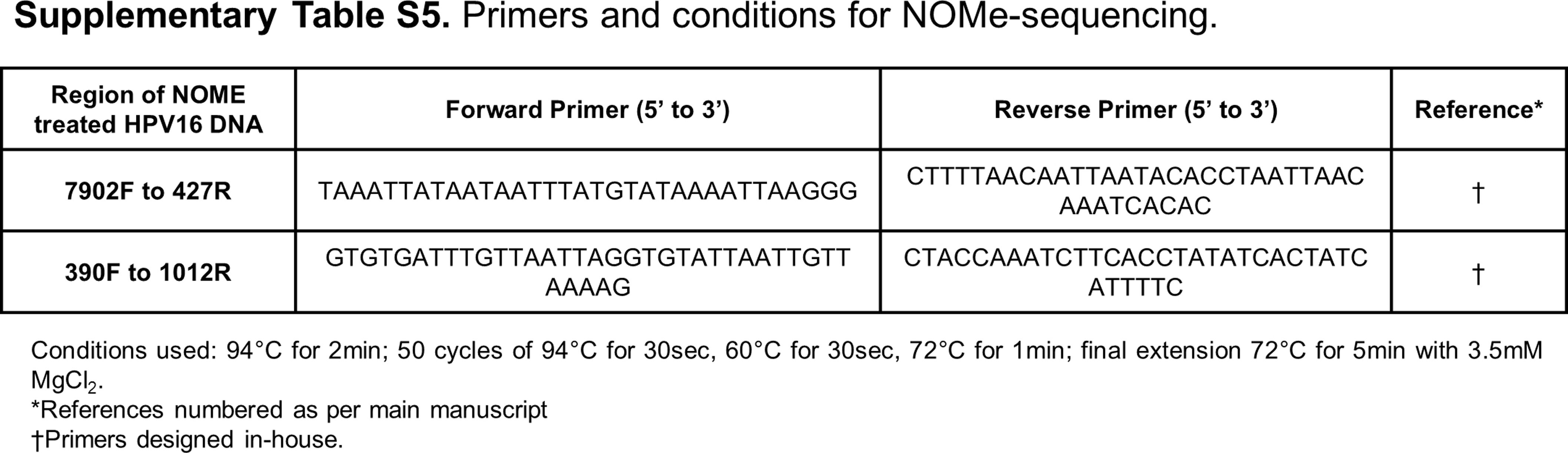

Supplement: Supplementary Table 5 [file onc20168x10.tif]

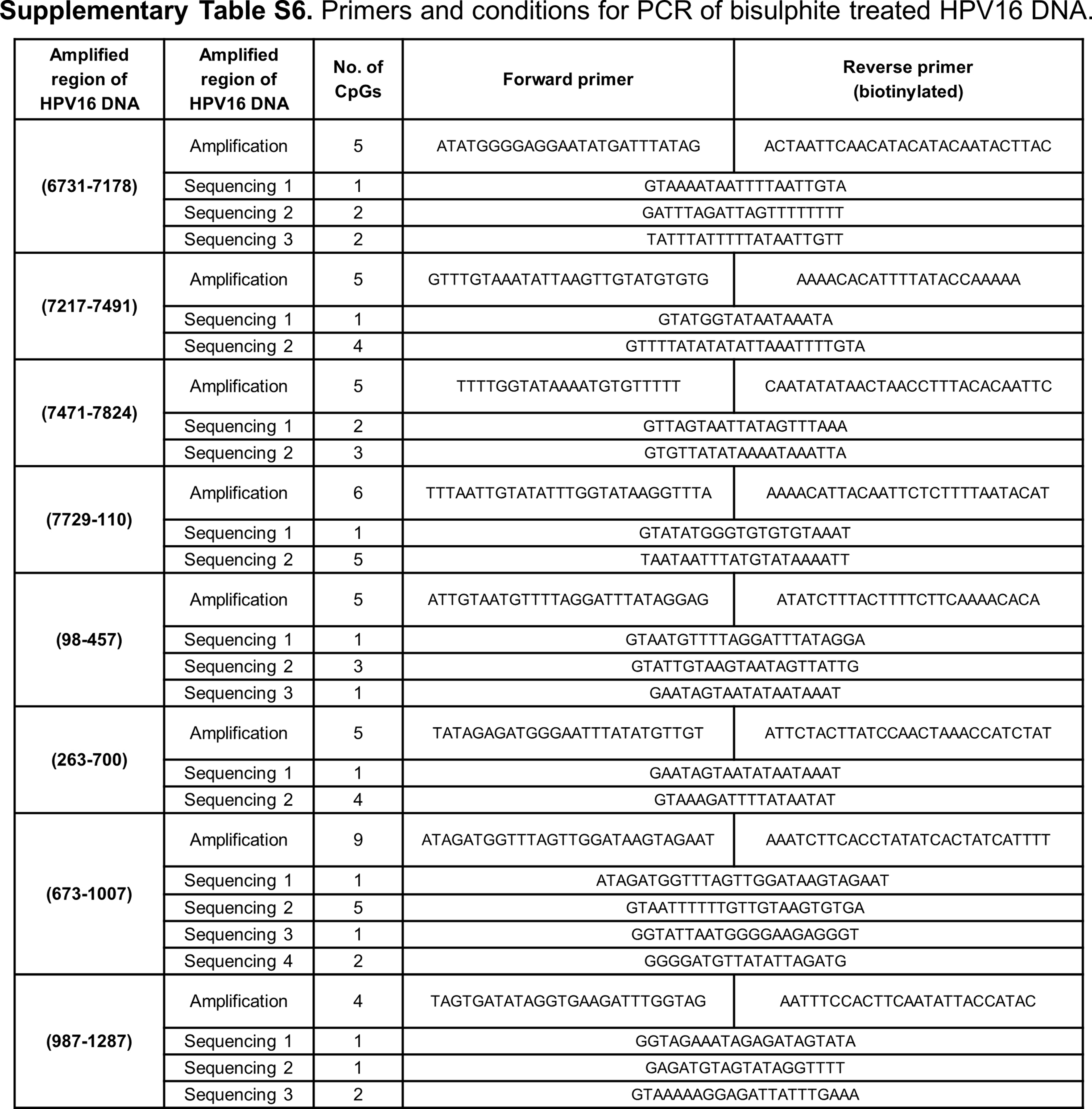

Supplement: Supplementary Table 6 [file onc20168x11.tif]
